# 

**Published:** 1912-01-15

**Authors:** 


### SUBJECT INDEX TO VOLUME LXVII.

EVENT AND COMMENT.

Advancing Standards, 315.

American Dental Journal, 418.

Cement Situation, 4.

Cleaning Teeth, 365.

Commercial Compensations and Professional Rewards, 157.

Editor Bethel, 105.

Excess in Eating, 207.

Financial Investments, 467.

How to Educate our Specialists, 261.

Independent Dental Journalism, 417.

Institute of Dental Pedagogics, 106.

National Dental Associations New Constitution, 573.

Nerve Supply of the Dentine, 365.

New Jersey Honors Meeker, 522.

Ohio State Dental Society's New Move, 521.

Ohio State Meeting, 576.

Professional Ranking, 313.

Reorganization of the National Dental Association, 3, 310.

Safford G. Perry, Dead, 1.

The 1912 Dental Journals, 54.

The Passing of Men of Mark, 53.

Taggart Patent Suit Revived, 1.

Taggart Suit, 106.

Uniformity in State Board Examinations, 578.

Uniformity in Professional Laws, 578.

Wright, C. M., Dead, 2.

FORMAL PAPERS.

An Attempt to Correct a few Errors, Henry W. Morgan, 579.

A Wholesale Cleaner, Oscar Dowling, M.D., 25.

Advice to Beginners, Geo. W. Bowling, 160.

Asepsis, Wanted a Sense of, J. S. Marshall, 399.

Bacterial Therapy, W. O. Wetmore, M.D., 488.

Care of the Hands, R. St. J. Perry, 145.

Carious Teeth as Carriers of Infection, T. E. Turner, 295.

Casting Plates by Campbell's Cow Bell, D. B. Campbell, 41.

Cleansing Root Canals, A. W. Ward, 4.

Dental Caries Caused by Flours from which the Enzymes have been Extracted, Thos. G. Read, L.D.S., 288.

Dental Economics, E. S. Coyne, 76

Dentistry Fifty Years Ago, J. P. Root, 451.

Distribution of the Nerves of the Dental Pulp, J. Howard Mummery, L.D.S., 342, 367.

Drug Idolatry in Dental Medicines, H. Prinz, 30.

Economic Value of Deciduous Teeth, Evangeline Jordan, 269.

Education of Patients at the Chair, G. R. Warner, 89.

Educating the Dentist, A. E. Royce, 428.

Guaranteed under the Food and Drug Act, J. U. Lloyd, M.D., 332.

Harmful Oral Bacilli and their Relation to Dental Caries, Theo. von Buest, 442.

Higher Efficiency in Teaching and Practicing Dentistry, C. N. Johnson, 59.

How to keep Well, W. A. Evans, 456.

Ignorance and Superstition Factors in the Treatment of School Children, Jacob Sobel, M.D., 444.

Impression Making, N. S. Hoff, 318.

Infected Area around the Ends of the Roots, M. L. Rhein, 419.

Influence of Diseased Mouths on Individual and Community Health, H. W. Starkey, M.D., 276.

Is the Study of Biology of Advantage to the Practicing Dentists C. M. Wright, 80.

International Dental Literature Index, 339.

Investments, C. G. Deaves, 334.

Lead in the Treatment of Perforated Roots, J. H. Spaulding, 484.

Local Anesthetics, T. I. Ottesen, 547.

Medical Education in Europe, 405.

Metabolism of the Enamel with Reference to it's Resistance to Caries, Theo. von Buest, 496.

Modern Methods of Producing Anesthesia, H. Prinz, 112.

Nearing Dental Anesthesia, 556.

Necrosis of the Alveolar Edge by Tincture of Iodine, Dr. Nux, 460.

New Apparatus for Administering Anesthesia, R. C. Coburn, M.D., 165.

Nitrous Oxide and Oxygen Anesthesia, E. S. Barber, 390.

Oral Hygiene Problem, G. E. Hunt, 124.

Oxyphosphate Cement Situation, W. V. B. Ames, 7.

Panama Pacific Dental Congress, F. L. Platt, 500.

Peridental Anesthesia, Frank L. Platt, 602.

Physiologic Features of High Altitudes, 502.

Plates versus Bridges, S. S. Doran, L.D.S., 133.

Points of Interest in Oral Surgery, J A. Gardner, D.D.S., 587.

Practical Prosthetic Casting, F. E. Roach, 477.

President's Address, C. A. Tavel, 523.

Prevention in Dentistry Today, J. M. Howe, 471.

Prevention of Shock and Vomiting in Chloroform Anesthesia, E. Stuver, M.D., 329.

Professional Schools, 462.

Prophylaxis of Maxillary Development, N. S. Hoff, 216.

Prophylaxis of Second Dentition, N. S. Hoff, 263.

Psychology of Pain, W. B. Pillsbury, 211.

Pyorrhea and Prophylaxis, F. H. Skinner, 171.

Relations of the Medical and Technical in Dental Education, V. Guerini, 137.

Refitting and Repairing Rubber Plates, J. L. Mewburn, 542.

Restoration by the Use of Silicate Cements, C. O. Rhea, 530.

School of the Job, W. F. Litch, 322.

State Boards versus Incompetent Dental Colleges, Geo. W. Bowling, 56.

Sterilization in Dental Practice, L. E. Custer, 187.

Story of the Mother and the Big Fat Hog, J. N. Hurty, M.D., 40.

Teeth and the Skull, 403.

The Childs Mouth from the Standpoint of the Physician and Dentist, J. E. Hunt, M.D., 493.

The Dentist the Guardian of the Gateway of Nutrition, S. M. Stauffer, 227.

The Technique of Making a Porcelain Jacket Crown by Pressure, J. O. Eppright, 498.

Toothache and Neuralgia, E. Tromner, 340.

The Porcelain Inlay, N. S. Jenkins, 326.

Treatment of Deciduous Dentition, N. S. Hoff, 107.

Tuberculosis from a Dental Standpoint, A. A. McLanahan, 533.

Unclean Mouth and its Evil Results, M. H. Fletcher, 382.

What shall we do to be Saved? Wm. W. Belcher, 68.

Why is a Dentist? C. L. Hungerford, 241.

OBITUARY.

Wm. H. Gilbert, 202

Joseph Root, 508.

Charles Stockton, 509.

C. M. Wright, 43.

BIBLIOGRAPHY.

American Text Book of Operative Dentistry, 96.

Buckley's Materia Medica, 98.

Wilson's Dental Prosthesis, 95.

Goepp's State Board Questions and Answers, 247.

Gould's Medical Dictionary, 191.

Irwin's Dental Laws, New Edition, 207.

The Mechanical Side of Articulation, 192.

Mikel's Dental Jurisprudence, 559.

Mouth Hygiene and Mouth Sepsis, 305.

Noyes Dental Histology, 408.

McCurdy's Oral Surgery, 344.

Prevention of Dental Caries and Oral Sepsis, 506.

Stedman's Medical Dictionary, 558.

INDENTS.

Age an Etiologic Factor in Pyorrhea, 567.

A Laboratory Help, 252.

Alopecia, 359.

Alveolar Hemmorrhage, 353.

Anesthesia in Treating Inflammation, 410.

A Question of Ethics, 201.

Arsenical Necrosis, 411.

A Spartan System of Hygiene, 101.

A Suggestion about Base Plates, 252.

A Valuable Root Filling, 304.

Avoiding Excess Water in Cast Inlay Investment, 51.

Bacteria of the Mouth and Value of Brushing and Dentifrices, 252.

Band Root Abutments, 616.

Bridges, 198.

California in 1915, 609

Canadian Oral Prophylactic Association, 346.

Care of the Tooth Brush, 252.

Casting against Pick Ups, 412.

Cast Gold Inlays, 251.

Cast Metal Appliances, 352.

Charcoal in Internal Diseases, 412.

Chicago's Tuberculosis Sanitarium, 154.

Cleaning Nerve Broaches, 302.

Cocain or Substitutes in Dental Operations, 100.

Cold Storage and Health, 355.

Compressed Oxygen, 47.

Controlling Hemorrhage in Setting Crowns, 303.

Controlling Hemorrhage, 49.

Cosmetology, 563.

Cotton, 253.

Crown and Bridges, 249.

Crown Post, 50.

Cutting Tools of a New Alloy, 514.

Dangers of Prophylaxis of Childrens Diseases, 515.

Dental Reserve Corps, 609.

Dentifrice, 50.

Disinfecting a Tooth with a Dead Pulp, 301.

Dont's in Pulp Anesthesia, 302.

Enlarging Pulp Canals, 49.

Essentials in Business, 197.

Ether Anesthesia by Venous Administration, 358.

Faith in the Dentists, 513.

Fees, 248.

Fighting Tuberculosis by Open Air Rooms, 613.

Foreign Students, 250.

Formaldehyde in Dentifrices, 353

Hope for the Elderly, 46.

House Fly and Bed Bug, 354.

Incidents of Office Practice, 200.

Incorporating Metals in Wax Models, 468.

Infant Nursing, how often, 516.

Inefficiency, a Reason for, 348.

Influence of Mastication on the Teeth, 565.

Infusion Anesthesia, 347.

Inhalone, 413.

Inlay Work, A Hint, 349.

Iodine in a Mouth Wash, 561.

Just Fees, 302.

Keep Tooth Brush Clean, 305.

Lanolin for the Hands, 612.

Liquid Celluloid, 302.

Medal for Humanitarian Service, 516.

Medical Value of Fruit, 609.

Mending Broken Plaster Models, 49.

Model Varnish, 49.

Nerves in Dentine, 350.

Neuralgia, 560.

New Electrical Discovery, 199.

New Metal, 249.

New Remedy for Burns, 208.

Nitrous Oxide Anesthesia, 303.

Obtaining a Smooth Palatal Surface in Vulcanite Plates, 565.

Open Air Rooms, 613.

Open Air Schools, 193.

Opening Teeth Devitalized with Arsenic, 562.

Oral Hygiene, 514.

Oral Prophylaxis, 560.

Our Greatest Asset, 615.

Owre some Walker, 249.

Pain, 514.

Partial Impression with Wax and Plaster, 50.

Perfect Fitting Clasps, 251.

Perfectly Fitting Porcelain Inlay, 354.

Period of Susceptibility to Dental Caries, 154.

Plate Polishing, 349.

Practice of Medicine and Dentistry in the Canal Zone, 512.

Preparing Sensitive Teeth for Crowns, 306.

Professional Attitude, 201.

Protecting Metal Plates during Vulcanizing, 353.

Protecting Gum Margins, 351.

Public Estimate of Dentistry, 193.

Phylosophy, Science and Medicine 568.

Quackery Abroad, 466.

Recovering Swallowed Foreign Bodies, 517.

Remedy for Burns, 51.

Removing Modeling Compound from Trays, 253.

Repairing Vulcanite Dentures, 199

Restoring Clogged Carborundum Wheels, 565.

Seal the Remedy, 196.

Separating Compound Impressions, 248.

Silicate Cements, Packing, 563.

Silicate Cements, Filling Instruments, 358.

Soldering a Richmond Crown, 350.

Solder Stick, 301.

Some Systemic Causes of Pyorrhea, 566.

Somnoform, 350.

Sterilizing Instruments, 197.

Symptoms of Nasal Obstruction, 352.

Take your Own Medicine, 565.

Technical versus Scientific, 102.

The Blood Pressure in Anesthesia, 250.

The Last Straw,—the Medical Nurse Doctor, 511.

The Miller Prize Awarded, 609.

The Next Dental Congress, 511.

Tongue and Buccal Cavity in Diagnosis, 194.

Tooth Paste Formula, 200.

Turpentine as a Lubricant, 51.

Ward's Inlay Investment Material 357.

Women in Dentistry, 467.

### ANNOUNCEMENTS.

American Society of Orthodontists 307.

Arizona Dental Society Officers, 52, 570.

Arkansas State Board of Examiners, 257.

Chapin A. Harris Memorial Fund, 103.

Chicago Dental Society, 616.

Chicago Dental Society to Honor Dr. Brophy, 520.

Clinic of National Dental Association, 254, 360.

Colorado State Dental Association, 204.

Congress of Hygiene and Demography, 361.

Correction, 570.

Ebers Papyrus, 155.

Evan's Museum and University of Pennsylvania, 519.

Georgia State Dental Society, 258.

Idaho State Board of Examiners, 307.

Illinois State Dental Society, 52, 208.

Indiana State Dental Society, 207, 258, 570.

Institute of Dental Pedagogics, 204, 520, 616.

International Congress of Hygiene and Demography, 309.

Kansas State Dental Society, 104.

Kentucky State Dental Association, 155, 208, 258.

Manufacturers Exhibit, 156.

Michigan State Board of Examiners, 206, 258.

Miller Memorial Fund, 202.

Minneapolis Dental Society, 570, 571.

Minnesota State Dental Association, 259.

Montana State Board of Examiners, 254.

National Association of Faculties, 360.

National Dental Association, 364, 414.

National Dental Protective Association, 260.

New Jersey State Dental Society, 205, 308.

North Carolina State Board of Examiners, 308.

North Dakota State Board of Examiners, 307.

Northern Ohio Dental Association, 307.

New York Dental Manufacturers Exhibit, 256.

New York Seventh and Eight District Societies, 518.

Ohio State Dental Society, 360, 571.

Oral Hygiene Society of Oklahoma, 253.

Panama Pacific Dental Congress, 255.

Present state of the Taggart vs. Boynton Suit, 362.

South Dakota State Dental Society, 103.

Southern New Jersey Dental Society, 260.

Tennessee State Dental Association, 156, 208, 259.

Tennessee Dental Association Officers, 52.

Texas State Board of Examiners, 259.

Texas State Dental Association, 156.

Vermont State Board of Examiners, 308.

Washington University Alumni Association, 204.

West Virginia State Dental Association, 307.

Wisconsin State Board of Examiners, 260.

AUTOGRAPHIC.

Allen, C. E., 253.

Ames, W. V. B., 7.

Argue, J. E., 49.

Barber, E. S., 390.

Baker, R. C., 607.

Bartlet, W. M., 349.

Belcher, Wm. W., 68.

Bogue, E. A., 353.

Bonesfield, E. C., 252.

Bowling, Geo. W., 56, 160.

Buckley, J. P., 197.

Campbell, D. B., 41.

Carpenter, E. R., 437.

Case, C. S., 433.

Cattell, D. M., 540, 584.

Clipsham, P., 563.

Coburn, R. C., M.D., 165.

Cole, J. M., 546.

Cook, George W., 305, 437.

Coyne, N. S., 76.

Crossland, J. H., 202.

Custer, L. E., 187.

Deaves, C. G., 334.

Doran, S. S., 133.

Dowling, Oscar, M.D., 7.

Dunn, E., 301.

Dunn, J. A., 49.

Duschinsky, Dr., 100.

Entricken, H. L., 51.

Eppright, J. O., 498.

Evans, W. A., M.D., 456.

Faught, J. A., 353.

Finley, M. F., 362.

Flanagan, Dr., 102.

Fletcher, M. H., 382, 423.

Flood, D., 608.

Fox, C. E., 607.

Gardner, J. A., 587.

Gillespie, W. C., 587.

Gilmer, T. L., 425.

Glucksman, G., 518.

Gregory, Dr., 201.

Grieves, C. J., 423.

Guerini, Vincenzi, 137.

Hawkins, G. E., 49.

Herty, J. N., M.D., 40.

Hinkins, J. E., 438.

Hoff, N. S., 107, 216, 263, 318.

Holbrook, C. W. F., 200.

Holder, Dr., 545.

Humm, P. S., 251.

Hungerford, C. L., 241.

Hunt, Geo. E., 124.

Hunt, J. E., M.D., 493.

Jacobson, A. C., M.D., 511.

Jenkins, N. S., 326.

Johnson, C. N., 59, 347, 433.

Jordan, Evangeline, 269.

Kellogg, Dr., 199.

Kester, J. P., 435.

Lane, J. G., 249.

Litch, W. F., 322.

Lloyd, J. U., 332.

Lowenthal, W., 349.

Lundy, E. W., 538.

McAbury, E. G., 51.

McCurdy, S. L., 425.

McLanahan, A. A., 533.

Mapps, Dr., 201.

Marshall, J. S., 399.

Martinier, P., 352.

Mewburn, J. L., 542, 546.

Morgan, H. W., 579.

Mummery, J. H., 342, 367.

Neal, Ewel, 530.

Neals, J., 306.

Noel L. G., 586.

Nux, Dr., 460.

Ottesen, T. I., 547.

Perry, R. St. J., 145.

Pillsbury, W. B., 211.

Platt, F. L., 500-602.

Porter, I. L., 349, 350.

Prinz, H., 30, 112.

Pupin, M. I., 199.

Read, Thos. G., 288.

Reid, J. G., 440.

Rhea, C. O., 530.

Rhein, M. L., 419, 420, 515.

Richards, Dr., 541.

DeRiemer, A. E., 351.

Riethmuller, R. H., 358.

Roach, F. E., 411, 477.

Root, J. P., 451, 561.

Royce, A. E., 428, 441.

Schamberg, M. I., 424.

Schmitz, M. L., 353.

Skinner, F. H., 171.

Smedley, V. C., 302.

Smith, A. G., 468.

Smith, Geo. E., 51.

Sobel, Jacob, M.D., 444.

Spaulding, J. H., 484.

Starkey, H. W., M.D., 276.

Stauffer, S. M., 227.

Stowell, C. F. B., 304.

Stuver, E., M.D., 329.

Sursen, R., 302.

Taggart, W. H., 412.

Talbott, F. S., 426.

Tavel, C. A., 523.

Torrance, C. M., 252, 354.

Tromner, E., 340.

Trueman, W. H., 563.

Turner, T. E., 295.

Von Buest, Theo., 442, 496.

### ANNOUNCEMENTS.

ILLINOIS STATE DENTAL SOCIETY. The forty-eighth annual meeting of the Illinois State Dental Society will be held at Springfield, May 14, 15, 16, 17, 1912.

A. E. CONVERSE, Chairman

Local Arrangements

Committe.

J. F. F. WALTZ, Secy.,

Decatur.

---

OFFICERS ARIZONA DENTAL SOCIETY. President, W. P. Sims; Vice-President, W. A. Baker; Secretary-Treasurer, H. H. Wilson; The society held a three days session in Phoenix November 7 to 9th, 1911.

---

OFFICERS TENNESSEE STATE DENTAL ASSOCIATION.—At the annual meeting in Memphis May 23—25, 1911, the following officers were elected: Charles A. Tavel, President; F. W. Meacham, First Vice-Pres.; W. G. Gillespie, Second Vice-Pres.; J. L. Manire, Recording Secretary; C. O. Rhea, Cor. Secretary; W. G. Hutchinson, Treasurer. The next place of meeting will be in Memphis.

